# Sepsis-assoziierte Enzephalopathie: Eine bundesweite Umfrage zu Verfahren der Diagnostik und des Neuromonitorings auf deutschen Intensivstationen

**DOI:** 10.1007/s00101-020-00853-z

**Published:** 2020-09-24

**Authors:** F. Klawitter, M. Jager, G. Klinkmann, T. Saller, M. Söhle, F. von Möllendorff, D. A Reuter, J. Ehler

**Affiliations:** 1 Klinik und Poliklinik für Anästhesiologie und Intensivtherapie, Universitätsmedizin Rostock, Schillingallee 35, 18057 Rostock, Deutschland; 2 Klinik für Anästhesiologie, Ludwig-Maximilians-Universität München, München, Deutschland; 3 Klinik für Anästhesiologie und Operative Intensivmedizin, Universitätsklinikum Bonn, Bonn, Deutschland

**Keywords:** Systemische Inflammation, Hirnschädigung, Kritische Erkrankung, Delirium, Umfragen und Fragebogen, Systemic inflammation, Brain injury, Critical illness, Delirium, Surveys and questionnaires

## Abstract

**Hintergrund:**

Die Sepsis-assoziierte Enzephalopathie (SAE) stellt eine der häufigsten Ursachen für eine neurokognitive Störung beim Intensivpatienten dar. Bisher existieren keine einheitlichen, evidenzbasierten Empfehlungen zum diagnostischen Vorgehen bei SAE.

**Ziel der Arbeit:**

Ziel der Studie ist die Evaluation des derzeitigen Vorgehens bei der Diagnostik und dem Neuromonitoring bei Patienten mit SAE auf deutschen Intensivstationen.

**Material und Methoden:**

In einer Online-Umfrage wurden 438 Mitglieder des Wissenschaftlichen Arbeitskreises Intensivmedizin (WAKI) und des Wissenschaftlichen Arbeitskreises Neuroanästhesie (WAKNA) befragt.

**Ergebnisse:**

Es konnten die Fragebögen von insgesamt 12,6 % aller Befragten ausgewertet werden (55/438). Ein standardisiertes diagnostisches Vorgehen bei SAE wurde von 21,8 % (12/55) der Befragten angegeben. Zur Detektion der SAE werden hauptsächlich Delir-Assessments (50/55; 90,9 %) und die klinische Untersuchung (49/55; 89,1 %) angewendet. Seltener werden die zerebrale Bildgebung (26/55; 47,3 %), Labor‑/Biomarkerbestimmungen (15/55; 27,3 %), elektrophysiologische Verfahren (14/55; 25,5 %) und Liquoruntersuchungen (12/55; 21,8 %) eingesetzt. Zur Verlaufskontrolle werden ebenfalls klinische Untersuchungen (45/55; 81,8 %) von den Befragten präferiert. Bei apparativen Neuromonitoring-Verfahren zeigen sich signifikante Unterschiede in theoretisch beigemessener Bedeutung und klinischer Anwendungspraxis. Die Mehrheit der Befragten (48/55; 87,3 %) befürwortet die Erstellung einheitlicher Empfehlungen für die Diagnostik und das Neuromonitoring bei SAE.

**Diskussion:**

Diese explorative Umfrage zeigt, dass bisher kein einheitliches Konzept im Hinblick auf Diagnostik und Neuromonitoring bei SAE auf deutschen Intensivstationen vorliegt. Innovative Biomarker der Hirnschädigung und apparative Neuromonitoring-Verfahren könnten in Zukunft die Diagnostik erleichtern und wertvolle prognostische Informationen zum neurokognitiven Outcome der Patienten mit SAE liefern.

**Zusatzmaterial online:**

Zusätzliche Informationen sind in der Online-Version dieses Artikels (10.1007/s00101-020-00853-z) enthalten. Die Online-Version dieses Beitrags (10.1007/s00101-020-00853-z) enthält den der Studie zugrunde liegenden Fragebogen. Beitrag und Zusatzmaterial stehen Ihnen auf www.springermedizin.de zur Verfügung. Bitte geben Sie dort den Beitragstitel in die Suche ein, das Zusatzmaterial finden Sie beim Beitrag unter „Ergänzende Inhalte“.

## Abstract (en)

**Background:**

Sepsis-associated encephalopathy (SAE) is one of the most frequent causes of neurocognitive impairment in intensive care patients. It is associated with increased hospital mortality and poor long-term neurocognitive outcome. To date there are no evidence-based recommendations for the diagnostics and neuromonitoring of SAE.

**Objective:**

The aim of the study was to evaluate the current clinical practice of diagnostics and neuromonitoring of SAE on intensive care units (ICU) in Germany.

**Material and methods:**

Based on available literature focusing on SAE, a questionnaire consisting of 26 items was designed and forwarded to 438 members of the Scientific Working Group for Intensive Care Medicine (WAKI) and the Scientific Working Group for Neuroanesthesia (WAKNA) as an online survey.

**Results:**

The total participation rate in the survey was 12.6% (55/438). A standardized diagnostic procedure of SAE was reported by 21.8% (12/55) of the participants. The majority of participants preferred delirium screening tools (50/55; 90.9%) and the clinical examination (49/55; 89.1%) to detect SAE. Brain imaging (26/55; 47.3%), laboratory/biomarker determination (15/55; 27.3%), electrophysiological techniques (14/55; 25.5%) and cerebrospinal fluid examination (12/55; 21.8%) are less frequently performed. The follow-up examination of SAE is most frequently performed by a clinical examination (45/55; 81.8%). Neuromonitoring techniques, such as continuous electroencephalography (31/55; 56.4%), transcranial doppler sonography (31/55; 56.4%) and near-infrared spectroscopy (18/55, 32.7%) are not frequently used. We observed statistically significant differences between the theoretically attributed importance and clinical practice. The great majority of respondents (48/55; 87.3%) endorse the development of guidelines containing recommendations for diagnostics and neuromonitoring in SAE.

**Discussion:**

This explorative survey demonstrated a great heterogeneity in diagnostics and neuromonitoring of SAE in German ICUs. Uniform concepts have not yet been established but are desired by the majority of study participants. Innovative biomarkers of neuroaxonal injury in blood and cerebrospinal fluid as well as electrophysiological and brain imaging techniques could provide valuable prognostic information on the neurocognitive outcome of patients and would thus be a useful addition to the clinical assessment of ICU patients with SAE.

## Hinführung

Die Sepsis-assoziierte Enzephalopathie (SAE) ist die häufigste neurologische Komplikation im Rahmen einer Sepsis und kann mit einem schlechten neurokognitiven Outcome sowie einer erhöhten Letalität assoziiert sein. Obwohl diese Art des Organversagens eine häufige Komplikation darstellt, existieren weder zur Diagnostik noch zum weiteren Neuromonitoring einheitliche Empfehlungen für die Intensivtherapie. Daher erfasst die vorliegende explorative Umfrage das gegenwärtige diagnostische Vorgehen bei Patienten mit SAE auf deutschen Intensivstationen.

## Hintergrund

Die SAE stellt eine multifaktoriell bedingte Dysfunktion des Zentralnervensystems (ZNS) im Rahmen einer Sepsis dar, welche 9–70 % der Sepsispatienten betrifft und mit einer erhöhten Letalität assoziiert ist [1]. Klinisch präsentiert sich dieses Syndrom sowohl mit akuten quantitativen (z. B. Somnolenz, Koma) als auch mit qualitativen (z. B. Agitation, Desorientiertheit) Störungen der Hirnfunktion und kann mit langfristigen neurokognitiven Schädigungen assoziiert sein [2]. Pathophysiologisch wird u. a. von einer Störung der Marko- und Mikrozirkulation mit Verlust der zerebralen Autoregulation (CA) ausgegangen, wodurch das Risiko zerebraler Ischämien oder Ödeme steigt [3, 4]. Diskutiert werden u. a. auch sekundäre Inflammationsreaktionen, die über aktivierte Astroglia eine direkte neuronale Schädigung hervorrufen können [5, 6].

Bisher wurden verschiedene diagnostische Ansätze, wie elektrophysiologische Verfahren (Elektroenzephalografie, EEG), zerebrale Bildgebungen (kranielle Computertomographie, cCT; kranielle Magnetresonanztomographie, cMRT), Messung von zerebraler Perfusion und Oxygenierung (transkranielle Dopplersonographie, TCD; Nahinfrarotspektroskopie, NIRS) sowie Biomarker in Blut oder Liquor bei Intensivpatienten mit SAE untersucht [1, 5, 7–10].

Bis auf einzelne Übersichtsartikel sind bisher keine einheitlichen Empfehlungen zum diagnostischen Vorgehen bei SAE verfügbar, sodass die Diagnosestellung und das Monitoring an die Erfahrungen der jeweiligen behandelnden Einrichtungen gebunden sind. Zur Evaluation der aktuellen intensivmedizinischen Praxis von Diagnostik und Neuromonitoring bei SAE auf Intensivstationen in Deutschland, wurde daher eine bundesweite explorative Umfrage durchgeführt. Die Autoren vermuten, dass es zum aktuellen Zeitpunkt deutschlandweit Unterschiede zwischen den verwendeten diagnostischen Methoden sowie deren Einsatzhäufigkeit gibt und dass die Bewertung der einzelnen diagnostischen Verfahren im Hinblick auf ihren Nutzen im Alltag uneinheitlich ist.

## Studiendesign und Untersuchungsmethoden

Für die Studie wurde ein Fragebogen mit 26 Fragen zu Art und Anwendungshäufigkeit verschiedener Diagnostika zur Detektion und zum Neuromonitoring bei SAE entwickelt. Die Erstellung, Verteilung und Auswertung des Online-Fragebogens (Zusatzmaterial online) erfolgte gemäß den gültigen Datenschutzrichtlinien und nach positivem Ethikvotum der Ethikkommission der Universität Rostock (Registriernummer A 2019-0001). Die Umfrage wurde mithilfe der Evaluationssoftware EvaSys® (Version 7.0, Electric Paper Evaluationssysteme GmbH, Lüneburg, Deutschland) erstellt. Die Mitglieder des Wissenschaftlichen Arbeitskreises Neuroanästhesie (WAKNA) und des Wissenschaftlichen Arbeitskreises Intensivmedizin (WAKI) der Deutschen Gesellschaft für Anästhesiologie und Intensivmedizin (DGAI) wurden per E‑Mail zur anonymen Umfrageteilnahme eingeladen. Doppelte Versendungen an Mitglieder beider Arbeitskreise wurden ausgeschlossen. Nachträgliche Rückschlüsse auf den jeweiligen Teilnehmenden waren nicht möglich. Einmalig wurde per E‑Mail zur Teilnahme an der Umfrage erinnert. Für die statistische Auswertung wurde die Software IBM SPSS Statistics (Version 25, IBM Corp., Armonk, NY, USA) verwendet. Unterschiede zwischen kategorialen Variablen wurden mit dem Chi-Quadrat-Test bzw. dem Exakten Test nach Fisher ermittelt. Statistische Signifikanz für einen Test wurde bei einem *p*-Wert <0,05 angenommen.

## Ergebnisse

### Allgemeine Basisdaten

Zwischen Februar und Mai 2019 wurde der Fragebogen an 438 Mitglieder des WAKI und WAKNA versandt und von 55 Mitgliedern (12,6 %) beantwortet (Tab. 1). Die Mehrzahl der Befragten ist als Chef- oder Oberarzt in einem universitären Haus der Maximalversorgung tätig. Alle Befragten (55/55; 100 %) hielten die SAE für eine relevante Komplikation. Bei 16,7 % (9/54) wurde 2018 eine klinikinterne Fortbildung zum Thema SAE durchgeführt. Eine Diagnostik zur Erfassung der SAE, egal welcher Art, führen 65,6 % (36/55) der Befragten auf ihren Intensivstationen durch, wobei 21,8 % (12/55) dafür eine hausinterne SOP nutzen.

**Table 1:** 

	Absolut (*n*)	Relativ (%)
**Verteilung nach Krankenhausversorgungsstufen**		
Grundversorgung	3	5,5
Schwerpunktversorgung	15	27,3
Maximalversorgung	7	12,7
Maximalversorgung (Universitätsklinik)	30	54,5
**Verteilung nach Klinik- und Weiterbildungsstatus**		
Klinikdirektor/Chefarzt	11	20,0
Oberarzt	39	70,9
Facharzt	4	7,3
Weiterbildungsassistent	1	1,8

### Sinnvolle Diagnostik bei Sepsis-assoziierter Enzephalopathie

**Figure 1:**
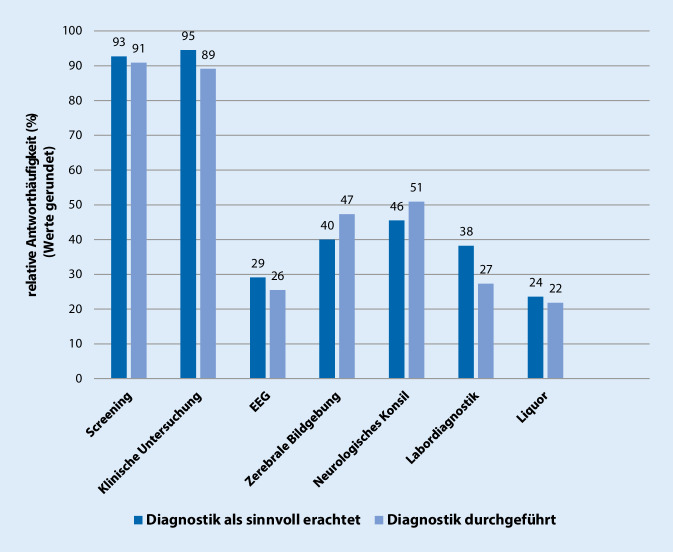

Die Verteilung der von den Befragten als sinnvoll erachteten diagnostischen Methoden bei SAE zeigt Abb. 1. Von einer Auswahl an vorgegebenen Diagnostika waren Delirscreeningverfahren (51/55; 92,7 %) und die klinische Untersuchung (52/55; 94,5 %) die am häufigsten als sinnvoll erachteten Diagnostika, wobei hier Mehrfachantworten möglich waren. Zur Detektion der SAE als Erstes Anwendung finden sollten gemäß 49,1 % (27/55) das Delirscreening und gemäß 40,0 % (22/55) die klinische Untersuchung. Apparative und laborchemische Untersuchungen wurden weniger häufig präferiert (EEG und neuropsychiatrisches Konsil jeweils 2/55; 3,6 %; Blut- und Liquordiagnostik jeweils 1/55; 1,8 %; zerebrale Bildgebung 0/55). Die eingesetzten Diagnostika würden die meisten Befragten (56,4 %, 31/55) nur bei konkretem Verdacht auf eine SAE durchführen (Tab. 2). Unter den Delirscreeningverfahren wird der CAM-ICU mit 68,5 % (37/54) präferiert. Das Screening soll gemäß 50,9 % (28/55) durch einen Arzt und gemäß 49,1 % (27/55) durch das Pflegepersonal durchgeführt werden. Die Anwendungshäufigkeit eines Screeningverfahrens wurde von 65,5 % der Befragten (36/55) mindestens einmal pro Schicht als angemessen angesehen (Tab. 2). Ein EEG sollte laut 43,1 % (22/51) als einmaliges Ruhe-EEG, laut 33,3 % (17/51) als mehrfaches Ruhe-EEG und laut 23,5 % (12/51) als kontinuierliches EEG zur SAE-Diagnostik eingesetzt werden. Bei der zerebralen Bildgebung sollte laut 66,7 % (36/54) zuerst eine cCT und laut 33,3 % (18/54) zuerst eine cMRT durchgeführt werden. Im Hinblick auf Biomarker wird die Bestimmung der neuronenspezifischen Enolase (NSE, 21/48; 43,8 %) am häufigsten als sinnvoll angesehen (Tab. 3). Die Hälfte der Befragten an Universitätsklinken hält, im Gegensatz zu einem Viertel der Befragten an Nichtuniversitätskliniken, die Labor- und/oder Biomarkerdiagnostik tendenziell für sinnvoller (15/30, 50 % vs. 6/25, 24 %; *p* = 0,057).

**Table 2:** 

	Absolut (*n*)	Relativ (%)
**Wie häufig erachten Sie es als sinnvoll, verschiedene Methoden zur Detektion der SAE einzusetzen?**		
Routinemäßig, bei jedem Patienten	23	41,8
Gelegentlich, wenn der Verdacht besteht	31	56,4
Selten, nur zur Ausschlussdiagnostik	1	1,8
**Wie häufig setzen Sie Diagnostik zur Detektion der SAE ein?**		
Routinemäßig, bei jedem Patienten	17	31,5
Gelegentlich, wenn der Verdacht besteht	33	61,1
Selten, nur zur Ausschlussdiagnostik	4	7,4
Gar nicht	0	0
**In welchen zeitlichen Abständen würden Sie ein Delir-Assessment (z.** **B. CAM-ICU) zur Erfassung der SAE durchführen?**		
Einmal pro Aufenthalt des Patienten	0	0
Einmal pro Tag	19	34,5
Einmal pro Schicht	36	65,5
**Wenn Sie EEG-Diagnostik bei SAE durchführen würden, wie häufig wäre dies?**		
Einmaliges Ruhe-EEG	22	43,1
Mehrfaches Ruhe-EEG in zeitlichen Abständen	17	33,3
Kontinuierliches EEG-Verfahren	12	23,5

**Table 3:** 

	Absolut (*n*)	Relativ (%)
Neuronenspezifische Enolase (NSE)	21	43,8
S100B-Protein	7	14,6
„Glial fibrillary acidic protein“ (GFAP)	5	10,4
Andere	15	31,3

### In der Praxis tatsächlich durchgeführte Diagnostik bei Sepsis-assoziierter Enzephalopathie

Die tatsächlich in der klinischen Praxis durchgeführten diagnostischen Methoden bei SAE zeigt Abb. 1. Delirscreenings (50/55; 90,9 %) sowie die klinische Untersuchung (49/55; 89,1 %) werden gegenüber anderen Diagnostika häufiger angewendet. An Universitätskliniken werden/wird tendenziell häufiger Labor- und/oder Biomarkerdiagnostik durchgeführt als an nichtuniversitären Einrichtungen (15/30 vs. 6/25; *p* = 0,057). Unabhängig von der Versorgungsstruktur des Krankenhauses werden Delirscreeningverfahren (27/55; 49,1 %) und die klinische Untersuchung (22/55; 40,0 %) am häufigsten als Verfahren der 1. Wahl angesehen. Die Majorität der Befragten führt Diagnostik erst bei konkretem Verdacht (33/54; 61,1 %) auf eine SAE und nicht routinemäßig zum Screening (17/54; 31,5 %) durch (Tab. 2).

### Neuromonitoring-Verfahren

**Figure 2:**
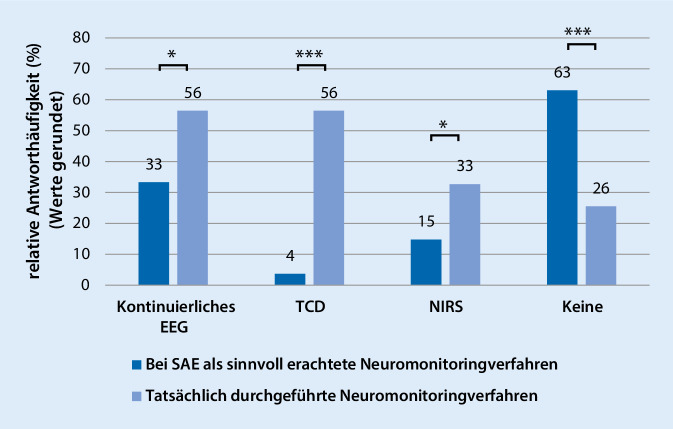

Insgesamt halten 81,8 % (45/55) es für sinnvoll, nach erfolgter Detektion der SAE, engmaschigere klinische Kontrollen abzuleiten (Abb. 2). Apparative Neuromonitoring-Verfahren halten 10,9 % (6/55) der Befragten für sinnvoll, wobei eine kontinuierliche EEG-Ableitung (18/54; 33,3 %) präferiert würde. Keine Änderung im Prozedere bei Patienten mit SAE hielten 7,3 % (4/55) für sinnvoll.

In der klinischen Praxis tatsächlich Anwendung finden verschiedene Neuromonitoring-Verfahren, wobei am häufigsten das EEG und die TCD (je 31/55; 56,4 %) eingesetzt werden. Universitätsklinken führen häufiger apparative Verfahren wie das EEG (Universitätskliniken: 21/30, 70,0 %; Nichtuniversitätskliniken: 10/25, 40,0 %; *p* = 0,03) und die NIRS (Universitätsklinken: 15/30, 50,0 %; Nichtuniversitätsklinken: 3/25, 12 %; *p* = 0,004) durch. Signifikante Unterschiede zeigen sich zwischen der theoretisch beigemessenen Bedeutung und der tatsächlichen klinischen Anwendungspraxis bei den einzelnen Neuromonitoring-Verfahren (Abb. 2). Neben der klinischen Patientenversorgung beschäftigen sich 16,7 % (9/54) der Befragten auch wissenschaftlich mit der Thematik SAE. Im Rahmen der Sepsis wird die SAE von 51,9 % (28/54) als eigene Organkomplikation erfasst, und von 56,4 % (31/55) werden die Angehörigen der Patienten über mögliche längerfristige neurokognitive Defizite bei SAE aufgeklärt.

Es sprechen sich insgesamt 87,3 % (48/55) für die Erstellung einer Leitlinie zum Thema SAE aus. Ein relevanter Unterschied in dieser Einschätzung besteht zwischen Universitätskliniken und Nichtuniversitätskliniken nicht (25/30, 83,3 % vs. 23/25, 92 %; *p* = 0,436) (Tab. 4).

**Table 4:** 

	Universitätskliniken		Nichtuniversitätskliniken		
	Absolut (*n*)	Relativ (%)	Absolut (*n*)	Relativ (%)	*p*-Wert
Kontinuierliche EEG-Überwachung	21	70,0	10	40,0	0,03
Transkranielle Dopplersonographie (TCD)	19	63,3	12	48,0	0,29
Nahinfrarotspektroskopie (NIRS)	15	50,0	3	12,0	0,004
Keine	5	16,7	9	36,0	0,13

## Diskussion

Die vorliegende Umfrage gibt erstmalig die aktuelle klinische Praxis von Diagnostik und Neuromonitoring bei SAE auf deutschen Intensivstationen wieder. Hierbei konnte gezeigt werden, dass die SAE als relevante intensivmedizinische Komplikation wahrgenommen wird, jedoch ein einheitlicher Standard für Diagnostik und Monitoring bisher nicht etabliert ist. In der aktuellen S3-Sepsisleitlinie wird zwar auf neurologische Komplikationen bei Sepsis verwiesen [11], eine evidenzbasierte Bewertung der bisher untersuchten Ansätze zu Diagnostik und Neuromonitoring bei SAE fehlt jedoch. Das Fehlen einer einheitlichen Nomenklatur und einer eindeutigen Definition von neurokognitiven Störungen bei einer Sepsis kommt erschwerend hinzu, weshalb ein erster wesentlicher Schritt zur Etablierung eines Standards die Verwendung einer einheitlichen Terminologie wäre [12]. Das strukturierte diagnostische Erfassen der SAE bei Patienten mit Sepsis stellt eine Grundvoraussetzung dar, um Präventions- oder Interventionsmöglichkeiten zu entwickeln, mit deren Hilfe zukünftig idealerweise das neurokognitive Outcome der Patienten sinnvoll beeinflusst werden kann.

### Diagnostische Verfahren zur Detektion der Sepsis-assoziierten Enzephalopathie

#### Klinischer Status und Delirscreeningverfahren

Neurologische Symptome können bei einer Sepsis innerhalb der ersten 48 h auftreten und frühzeitig in der klinischen Untersuchung bettseitig erfasst werden [13]. Hierbei ist jedoch zu beachten, dass es sich bei der SAE um eine Ausschlussdiagnose handelt und somit andere Entitäten mit ähnlicher klinischer Symptomatik, wie z. B. der ischämische Schlaganfall oder ein Status epilepticus, mittels der empfohlenen Diagnostik ausgeschlossen werden sollten [7].

Standardisierte Screening-Tools wie der CAM-ICU oder die Intensive Care Delirium Screening Checklist (ICDSC) sind für die Diagnosestellung des Delirs beim Intensivpatienten gut validiert [14]. Übereinstimmend zeigen die vorliegenden Ergebnisse, dass neben der klinischen Untersuchung die Delirscreeningverfahren häufiger als apparative oder laborchemische Untersuchungen angewendet werden. Sinnvoll kann jedoch gerade eine Kombination von mehreren Verfahren sein, da eine Beschränkung auf das reine Delirscreening zum Übersehen fokal-neurologischer Defizite, z. B. im Rahmen von Hirnischämien bei SAE, führen könnte [9, 15]. Zu beachten ist weiterhin, dass die in dieser Umfrage favorisierten Delir-Assessments (CAM-ICU: 37/74; 68,5 % und ICDSC: 7/54; 13 %) nicht speziell an septischen Intensivpatienten evaluiert wurden.

#### Apparative und laborchemische Verfahren zur Frühdiagnostik

Apparative und laborchemische Verfahren zur Detektion der SAE finden laut der vorliegenden Umfrage aktuell verhältnismäßig wenig Anwendung. Ein Vorteil bettseitiger Neuromonitoring-Verfahren könnte aber in der Beurteilung sedierter und klinisch nur schwer beurteilbarer Patienten bestehen. So konnten Schramm et al. in einer Studie mit 30 analgosedierten septischen Intensivpatienten durch Anwendung von TCD innerhalb der ersten 24 h bei 60 % der Patienten eine gestörte CA detektieren, was signifikant mit dem Auftreten einer SAE korrelierte [16]. Weiterhin konnte in EEG-Studien gezeigt werden, dass pathologische Veränderungen der elektrischen Hirnaktivität bei bis zu 32 % der erwachsenen und in bis zu 58 % der pädiatrischen Sepsispatienten erfasst werden können und mit einer erhöhten Letalität assoziiert sind [17]. Auch könnten Biomarker der neuroaxonalen Schädigung hilfreich sein, bettseitig die Detektion und Verlaufskontrolle der SAE zu ermöglichen. In einer Pilotstudie konnte eine positive Korrelation erhöhter Konzentrationen von Neurofilamentleichtketten im Plasma und dem Auftreten einer SAE nachgewiesen werden [10].

#### Zerebrale Bildgebung

Das Auftreten neuer zerebraler Läsionen im cMRT bei Sepsis ist mit einem schlechten neurokognitiven Outcome bis zu 12 Monate nach der Erkrankung assoziiert ist [15, 18]. Laut den Ergebnissen der vorliegenden Studie wird das cCT gegenüber einem cMRT als initiale Bildgebung von den Befragten favorisiert, was sich möglicherweise in der schnelleren Durchführbarkeit oder breiteren Verfügbarkeit des CT begründet. Allerdings sollte berücksichtigt werden, dass ein cMRT für die Detektion pathologischer Befunde eine höhere Sensitivität aufweist [19]. Limitierende Faktoren für eine routinemäßige Anwendung bildgebender Verfahren sind hierbei allerdings der logistische und zeitliche Aufwand sowie das erhöhte Komplikationsrisiko für Intensivpatienten im Rahmen des innerklinischen Transportes, was eine individuelle Nutzen-Risiko-Abwägung unabdingbar macht [20].

### Diagnostische Verfahren zur Verlaufskontrolle der Sepsis-assoziierten Enzephalopathie

Gemäß den hier vorliegenden Umfrageergebnissen findet bei SAE in der Praxis die klinische Verlaufskontrolle mehr Zustimmung als das apparative Neuromonitoring. Hier könnten sowohl die Geräteverfügbarkeit auf den jeweiligen Intensivstationen als auch die Erfahrungen in der technischen Anwendung und Befundauswertung ein aktuell noch limitierender Faktor sein.

#### Elektroenzephalographie

Eine kontinuierliche EEG-Überwachung könnte helfen, nichtkonvulsive epileptische Anfallsaktivität oder einen Status epilepticus zu erfassen und zu behandeln, da mit zunehmender Anfallsdauer die Mortalität nachweislich zunimmt [21]. Die Tatsache, dass 56,4 % (31/55) der Befragten angeben, ein EEG-Verfahren im Rahmen des Neuromonitorings einzusetzen und immerhin 33,3 % (18/54) dies auch bei der SAE als sinnvoll betrachten würden, spricht für eine partielle klinische Akzeptanz von EEG-Verfahren. Beachtet werden muss hierbei, dass in der vorliegenden Umfrage nicht differenziert nach der Art des angewendeten EEG-Verfahrens gefragt wurde, sodass hier sowohl das Mehrkanal-EEG als auch ein EEG-basiertes Monitoring-Verfahren (z. B. Bispektralindex, BIS) subsumiert sein können. Dass Universitätskliniken signifikant häufiger ein EEG einsetzten als Nichtuniversitätskliniken, könnte an der häufig stärker interdisziplinär geprägten Versorgungsstruktur an Universitätsklinken mit häufig angebundener Neurologie liegen. Des Weiteren könnte die Nutzung von Monitoring-Verfahren für Forschungsfragestellungen im Rahmen klinischer Studien einen relevanten Punkt darstellen, warum diese Verfahren häufiger Anwendung an Universitätskliniken im Vergleich zu Nichtuniversitätskliniken finden.

#### Transkranielle Dopplersonographie, Nahinfrarotspektroskopie und Monitoring der zerebralen Autoregulation

Unter Anwendung der TCD konnte bei Sepsispatienten überwiegend eine gestörte CA nachgewiesen werden, was mit dem Auftreten neurologischer Defizite korreliert [3, 4, 16, 22]. In der vorliegenden Umfrage gaben 56,4 % (31/55) der Befragten an, das TCD im Rahmen der neurointensivmedizinischen Diagnostik regelmäßig auf den jeweiligen Intensivstationen eingesetzt wird. Diskrepant dazu ist allerdings, dass lediglich 3,7 % (2/54) der Befragten den TCD als eine sinnvolle Diagnostik bei der SAE bewerteten. Dies steht im Gegensatz zur bisher vorhandenen Literatur und könnte möglicherweise ebenfalls an der Verfügbarkeit entsprechender Messsysteme auf den Intensivstationen sowie an Unsicherheiten über direkte therapeutische Konsequenzen der erhobenen Befunde liegen.

Nur wenige, nichtkontrollierte Studien mit geringen Fallzahlen haben bisher die regionale zerebrale Oxymetrie zur Beurteilung der CA im Rahmen einer SAE untersucht [23, 24]. Die bisherige Studienlage konnte bisher keinen sicheren Nutzen aufzeigen, da einige Arbeiten zerebrale Autoregulationsstörungen bei Sepsis durch die Anwendung von NIRS oder dem zerebralen Oxymetrieindex detektierten [23], während andere Arbeiten keine Relevanz für die Sepsis und SAE feststellen konnten [24].

#### Biomarker

Die Wertigkeit der Biomarkerdiagnostik bei der SAE wird in der vorliegenden Umfrage als gering eingeschätzt, da lediglich 27,3 % der Befragten Biomarker in der klinischen Praxis bestimmen. Im Hinblick auf die vorhandene Literatur wurde die NSE, neben dem S100B-Protein, am häufigsten evaluiert, wobei gezeigt werden konnte, dass gesteigerte Serumkonzentrationen sowohl mit einer Zunahme des Enzephalopathierisikos als auch mit einer erhöhten 30-Tage-Sterblichkeit korrelieren [25, 26]. Dem S100B-Protein wird hierbei eine höhere diagnostische Wertigkeit für die Detektion und Prognoseabschätzung eingeräumt [26]. Neurofilamente als Marker einer axonalen Schädigung bei SAE stellen einen neuartigen interessanten Ansatz in der Biomarkerdiagnostik dar, da eine positive Korrelation erhöhter Serumspiegel mit dem Ausmaß der kognitiven Dysfunktion und der Dreimonatssterblichkeit gezeigt werden konnte [10]. Zukünftig könnten auch geeignete Biomarker-Panels zur Differenzierung passagerer Hirnfunktionsstörungen und bleibender Hirnschädigungen bei SAE von Nutzen sein [5]. Diese Biomarker-gestützte Stufendiagnostik könnte insbesondere die Indikationsstellung zur Durchführung einer zerebralen Bildgebung erleichtern und somit helfen, das Ausmaß der Hirnschädigung und die neurokognitive Langzeitprognose der Patienten zu erfassen [5, 9, 15].

## Zusammenfassung

Mit dieser explorativen Umfrage kann gezeigt werden, dass eine methodische Heterogenität bei Diagnostik und Monitoring der SAE auf deutschen Intensivstationen vorliegt.

Für die Bewertung der Ergebnisse dieser Umfrage muss einschränkend bemerkt werden, dass die Umfragebeteiligung mit insgesamt 12,6 % aller angeschriebenen Kollegen insgesamt niedrig war, was bei der Interpretation der Ergebnisse berücksichtigt werden muss und die Generalisierbarkeit einschränkt. Dennoch existieren bisher keine Umfrageergebnisse zu diesem Thema, sodass insbesondere für intensivmedizinisch tätige Kollegen, die täglich mit der Versorgung von Patienten mit SAE betraut sind, ein Überblick zum aktuellen Stellenwert einzelner diagnostischer Verfahren gegeben werden kann. Weiterhin ist anzumerken, dass durch die gezielte Befragung von ausschließlich Mitgliedern des WAKI und WAKNA eine gewisse Selektion fachlich spezialisierter Kolleginnen und Kollegen erfolgte und ein „selection bias“ folglich nicht ausgeschlossen werden kann. Interessant wäre es zukünftig, auch Ärzte außerhalb dieser Arbeitskreise zu befragen, um ein umfassenderes Bild zur Diagnostik bei SAE zu bekommen. Auch wenn eine evidenzbasierte Bewertung einzelner diagnostischer Verfahren teilweise noch aussteht, befürwortet eine große Mehrheit der Befragten die Erstellung einer Leitlinie zum Thema Diagnostik und Neuromonitoring bei SAE, um ein einheitliches Vorgehen unter Nutzung der vorhandenen diagnostischen Verfahren zu ermöglichen. Die Bewertung dieser Verfahren sollte im Rahmen des Entstehungsprozesses einer SAE-Leitlinie erfolgen und der nächste Schritt bei diesem klinisch relevanten Thema sein.

## Fazit für die Praxis

- Ein einheitliches diagnostisches Konzept bei SAE (Sepsis-assoziierte Enzephalopathie) existiert auf vielen deutschen Intensivstationen bisher nicht.
- Sinnhaftigkeit und Nutzen einzelner diagnostischer Verfahren werden unterschiedlich bewertet, sodass uneinheitliche Anwendungskonzepte nachgewiesen werden können.
- Die Erstellung einheitlicher, evidenzbasierter Empfehlungen zu Diagnostik und zum Neuromonitoring, z. B. in Form einer Leitlinie, wird von der Mehrheit der Befragten befürwortet.

## Caption Electronic Supplementary Material


