# Supplementary material for: Sepsis-associated encephalopathy: A nationwide survey on diagnostic procedures and neuromonitoring in German intensive care units
Source: Anaesthesist. 2020 Sep 24;70(2):112–20. [Article in German] doi: 10.1007/s00101-020-00853-z (PMC7851101; doi:10.1007/s00101-020-00853-z)
Supplement: Supplementary file 1 [file 101_2020_853_MOESM1_ESM.pdf]

Zusatzmaterial zum Beitrag
Klawitter F., Jager M., Klinkmann G. et al- (2020) Sepsis-assoziierte Enzephalopathie. Anaesthesist.

Beitrag und Zusatzmaterial stehen Ihnen auf www.springermedizin.de zur Verfügung. Bitte geben Sie dort den Beitragstitel in die Suche ein

Universitätsmedizin Rostock
Klinik und Poliklinik für Anästhesiologie und Intensivtherapie
AG NeuroIntens

Sepsis-assoziierte Enzephalopathie

Eine bundesweite Umfrage zu Verfahren der Diagnostik und des Neuromonitorings auf deutschen Intensivstationen

1. Einwilligung zur Teilnahme

1.1 **Die Teilnahme an dieser Studie ist freiwillig. Ihre Daten werden anonym behandelt, sodass nach der Auswertung kein individueller Rückschluss möglich ist. Die Datenerhebung wird voraussichtlich bis Juni 2019 abgeschlossen sein.**

- ☐ Ja, ich bin einverstanden und möchte an dieser Umfrage teilnehmen.

2. Basisdaten

2.1 **In welchem Bundesland befindet sich Ihre Klinik?**

- ☐ Baden-Württemberg
- ☐ Bayern
- ☐ Berlin
- ☐ Brandenburg
- ☐ Bremen
- ☐ Hamburg
- ☐ Hessen
- ☐ Mecklenburg-Vorpommern
- ☐ Niedersachsen
- ☐ Nordrhein-Westfalen
- ☐ Rheinland-Pfalz
- ☐ Saarland
- ☐ Sachsen
- ☐ Sachsen-Anhalt
- ☐ Schleswig-Holstein
- ☐ Thüringen

2.2 **Bitte ordnen Sie Ihre Klinik in eine der folgenden Versorgungsstufen ein:**

- ☐ Grundversorgung
- ☐ Schwerpunktversorgung
- ☐ Maximalversorgung
- ☐ Maximalversorgung (Universitätsklinik)

2.3 **Welche Position haben Sie in Ihrem Klinikum?**

- ☐ Klinikdirektor / Chefarzt
- ☐ Oberarzt
- ☐ Facharzt
- ☐ Weiterbildungsassistent

2.4 **Halten Sie die SAE für eine relevante Komplikation beim Intensivpatienten?**

☐ Ja ☐ Nein

2.5 **Gab es bei Ihnen im vergangenen Jahr eine Fortbildung zum Thema SAE?**

☐ Ja ☐ Nein

2. **Basisdaten** [Fortsetzung]

2.6 **Wird auf Ihrer Intensivstation Diagnostik zur Erfassung der SAE durchgeführt?**

☐ Ja ☐ Nein

2.7 **Gibt es zum jetzigen Zeitpunkt auf Ihrer Intensivstation ein strukturiertes diagnostisches Vorgehen bei SAE (z.B. eine SOP)?**

☐ Ja ☐ Nein

2.8 **Wer stellt auf Ihrer Intensivstation in der Regel die Verdachtsdiagnose SAE?**

☐ Anästhesist ☐ Neurologe ☐ Chirurg

☐ Internist

3. Diagnostik der SAE

3.1 **Welche Methoden würden Sie für die Diagnostik der SAE als sinnvoll erachten?**

Bitte wählen Sie **alle zutreffenden** Antwortmöglichkeiten aus:

- ☐ **A** Screeningverfahren (z.B. Confusion Assessment Method for the Intensive Care Unit (CAM-ICU), Intensive Care Delirium Screening Checkliste (ICDSC))
- ☐ **B** Klinisch-intensivmedizinische Untersuchung
- ☐ **C** Elektroenzephalographie (EEG)
- ☐ **D** Zerebrale Bildgebung (z.B. Computertomographie (CT), Magnetresonanztomographie (MRT))
- ☐ **E** Neurologisches Konsil
- ☐ **F** Labordiagnostik und/oder Biomarkerbestimmung im Serum
- ☐ **G** Liquordiagnostik

3.2 **Wie häufig würden Sie die von Ihnen angegebenen Methoden zur Detektion der SAE einsetzen?**

- ☐ Routinemäßig, bei jedem Patienten
- ☐ Gelegentlich, wenn der Verdacht besteht
- ☐ Selten, nur zur Ausschlussdiagnostik

3.3 **Welche Methoden werden auf Ihrer Intensivstation zur Detektion der SAE eingesetzt?**

Bitte wählen Sie **alle zutreffenden** Antwortmöglichkeiten aus:

- ☐ **A** Screeningverfahren (z.B. Confusion Assessment Method for the Intensive Care Unit (CAM-ICU), Intensive Care Delirium Screening Checkliste (ICDSC))
- ☐ **B** Klinisch-intensivmedizinische Untersuchung
- ☐ **C** Elektroenzephalographie (EEG)
- ☐ **D** Zerebrale Bildgebung (z.B. Computertomographie (CT), Magnetresonanztomographie (MRT))
- ☐ **E** Neurologisches Konsil
- ☐ **F** Labordiagnostik und/oder Biomarkerbestimmung im Serum
- ☐ **G** Liquordiagnostik
- ☐ **H** Keine

3.4 **Wie häufig setzen Sie (die von Ihnen angegebenen) Methoden zur Detektion der SAE ein?**

- ☐ Routinemäßig, bei jedem Patienten
- ☐ Gelegentlich, wenn der Verdacht besteht
- ☐ Selten, nur zur Ausschlussdiagnostik
- ☐ Gar nicht

3. **Diagnostik der SAE** [Fortsetzung]

3.5 **Welches diagnostische Verfahren würden Sie zur Detektion einer SAE <u>zuerst</u> einsetzen?**

- ☐ Screeningverfahren (z.B. CAM-ICU, ICDSC)
- ☐ Klinisch-intensivmedizinische Untersuchung
- ☐ EEG
- ☐ Zerebrale Bildgebung (CT, MRT)
- ☐ Neurologisches Konsil
- ☐ Labordiagnostik und/oder Biomarkerbestimmung im Serum
- ☐ Liquordiagnostik

3.6 **Wenn Sie ein Screeningverfahren zur Detektion der SAE verwenden würden, um welches würde es sich handeln?**

- ☐ Richmond Agitation-Sedation Scale (RASS)
- ☐ Sedation-Agitation Scale (SAS)
- ☐ CAM-ICU (Confusion Assessment Method for the Intensive Care Unit)
- ☐ ICDSC (Intensive Care Delirium Screening Checklist)
- ☐ Nursing Delirium Screening Scale (Nu-Desc)
- ☐ Delirium Detection Score (DDS)
- ☐ Stanford Proxy Test for Delirium (S-PTD)
- ☐ Assessment test for delirium & cognitive impairment (4AT)

3.7 **Wer sollte aus Ihrer Sicht ein Screeningverfahren zur Detektion der SAE vorrangig durchführen?**

- ☐ Pflege
- ☐ Arzt

3.8 **In welchen zeitlichen Abständen würden Sie ein Screeningverfahren zur Erfassung einer SAE durchführen?**

- ☐ Einmal pro Aufenthalt des Patienten
- ☐ Einmal täglich
- ☐ Einmal pro Schicht

3.9 **Wenn Sie EEG-Diagnostik bei SAE durchführen würden, wie häufig wäre dies?**

- ☐ Einmaliges Ruhe-EEG
- ☐ Mehrfaches Ruhe-EEG in zeitlichen Abständen
- ☐ Kontinuierliches EEG-Verfahren

3.10 **Wenn Sie eine zerebrale Bildgebung zur Diagnostik der SAE durchführen würden, welche würden Sie am ehsten <u>zuerst</u> durchführen?**

- ☐ Kranielles CT
- ☐ Kranielles MRT

3. **Diagnostik der SAE** [Fortsetzung]

3.11 **Wenn Sie Biomarker zur Detektion der SAE verwenden würden, welche wären dies in der Regel?**

- ☐ Neuronen-spezifische Enolase (NSE)
- ☐ S100B
- ☐ Glial Fibrillary Acid Protein (GFAP)
- ☐ Andere

4. Weiterführende Maßnahmen

4.1 **Welche Konsequenzen bzw. Maßnahmen würden SIe aus den erhobenen diagnostischen Befunden für die weitere Intensivtherapie ableiten?**

☐ Beginn eines Neuromonitorings (z.B. EEG-Überwachung, Transkranielle Dopplersonographie (TCD), Nahinfrarotspektroskopie (NIRS))

☐ Engmaschigere klinisch-neurologische Verlaufskontrolle

☐ Keine Änderung

4.2 **Welche Neuromonitoringverfahren würden Sie bei SAE als sinnvoll erachten?**

Bitte wählen Sie **alle zutreffenden** Antwortmöglichkeiten aus:

☐ **A** Kontinuierliche EEG-Überwachung

☐ **B** Transkranielle Dopplersonographie (TCD)

☐ **C** Nahinfrarotspektroskopie (NIRS)

☐ **D** Keine

4.3 **Welche Neuromonitoringverfahren werden bei Ihnen auf der Intensivstation durchgeführt?**

Bitte wählen Sie **alle zutreffenden** Antwortmöglichkeiten aus:

☐ **A** Kontinuierliche EEG- Überwachung

☐ **B** Transkranielle Dopplersonographie (TCD)

☐ **C** Nahinfrarotspektroskopie (NIRS)

☐ **D** Keine

4.4 **Beschäftigen Sie sich an Ihrer Klinik forschungsmäßig mit dem Thema SAE?**

☐ Ja ☐ Nein

4.5 **Erfassen Sie die SAE neben der Sepsis als eigenständige Diagnose des Patienten?**

☐ Ja ☐ Nein

4.6 **Werden Angehörige des Patienten bei Detektion einer SAE über mögliche, auch langfristige neurokognitive Defizite aufgeklärt?**

☐ Ja ☐ Nein

4.7 **Würden Sie eine Leitlinie zur Diagnosesicherung der SAE sinnvoll finden?**

☐ Ja ☐ Nein

4. **Weiterführende Maßnahmen** [Fortsetzung]

4.8 **Haben Sie noch Anmerkungen?**
